# Effect of Quinoline on the Phospholipid Profile of *Curvularia lunata* and Its Microbial Detoxification

**DOI:** 10.3390/molecules27072081

**Published:** 2022-03-24

**Authors:** Aleksandra Felczak, Katarzyna Zawadzka, Przemysław Bernat, Marta Nowak-Lange, Katarzyna Lisowska

**Affiliations:** Department of Industrial Microbiology and Biotechnology, Faculty of Biology and Environmental Protection, University of Lodz, 12/16 Banacha Street, 90-237 Lodz, Poland; katarzyna.zawadzka@biol.uni.lodz.pl (K.Z.); przemyslaw.bernat@biol.uni.lodz.pl (P.B.); marta.nowak@biol.uni.lodz.pl (M.N.-L.); katarzyna.lisowska@biol.uni.lodz.pl (K.L.)

**Keywords:** quinoline, fungal degradation, detoxification, phospholipid profile

## Abstract

Quinoline is an N-heterocyclic compound commonly found in wastewater, especially that derived from coal processing, chemical, and pharmaceutical industries. In the present study, the microscopic fungus *Curvularia lunata* IM 4417, which is known to degrade various xenobiotics, was used. The aim of the research was to study the elimination of quinoline and its influence on fungal phospholipids, which are considered to be excellent indicators of environmental monitoring. Quinoline biodegradation products and phospholipid contents were analyzed using gas chromatography–mass spectrometry and liquid chromatography–tandem mass spectrometry. *C. lunata* IM 4417 degraded quinoline, which led to the formation of conjugates of glucose with hydroxylated derivatives of the compound. Toxicity tests (Artoxkit M and Microtox assay) indicated that the elimination of lower concentrations of quinoline was efficient and led to a reduction in sample toxicity. The presence of quinoline also significantly affected the profile of fatty acids and phospholipids. The addition of quinoline to a culture of *C. lunata* IM 4417 caused an increase in the content of phosphatidylcholine (PC) and a decrease in the amount of phosphatidylethanolamine (PE), two major structural lipids. Additionally, decreases in the contents of phosphatidylinositol (PI) and phosphatidylserine (PS), which are responsible for tolerance to toxic substances, cell viability, and signal transduction, were noted. Thus, it can be concluded that the presence of quinoline modifies the membrane composition, and this change may be an important indicator of the presence of N-heterocyclic compounds or other toxins in the environment.

## 1. Introduction

**Figure 1:**
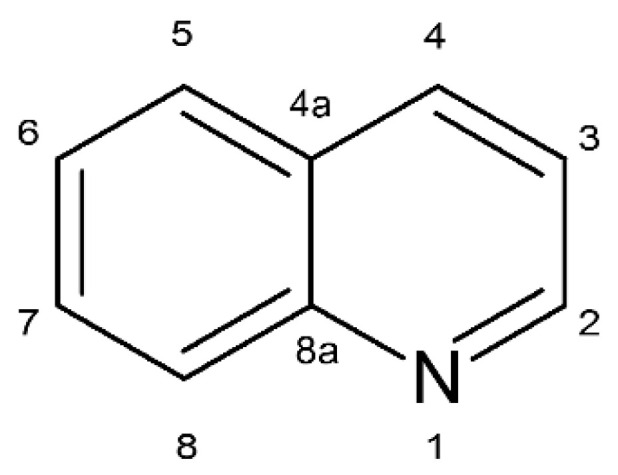
Chemical structure of quinoline.

Quinoline is an N-heterocyclic compound composed of a condensed benzene and pyridine ring and commonly occurs in coal tar, oil shale, crude oil, and petroleum (Figure 1). This compound is used as a raw material or solvent in the pharmaceutical, chemical, dye, and food industries [1,2,3].

Due to that fact, quinoline is commonly found in wastewater generated from the coal pyrolysis process, shale oil production, and drug manufacturing. Literature data indicate that quinoline concentration in semi-cooking wastewater is as high as 80 mg/L. High quinoline content has also been reported in soil and groundwater contaminated by creosote [4,5]. The relatively good solubility of quinoline in water, its low biodegradability, and widespread utilization have contributed to the contamination of diverse ecosystems. Moreover, the literature data indicate that quinoline is bioaccumulated by various organisms and can negatively affect different trophic levels. Its ecotoxicity has been demonstrated in tests using organisms such as *Aliivibrio fischeri*, *Pseudomonas putida*, *Daphnia magna*, *Desmodesmus subspicatus*, and *Danio rerio* [6,7,8]. Moreover, quinoline and its derivatives have been described to possess genotoxic and mutagenic activity [7]. Because quinoline and its derivatives are present in soil and water ecosystems, it is important to determine its impact on microorganisms which are typical for such habitats and estimate their ability to degrade quinoline, especially in low, environmental concentrations (up to 100 mg/L). Monitoring of intermediates formed during biodegradation and assessing their toxicity are equally important. 

In the literature, we can find relevant data addressing the bacterial degradation of quinoline, but little is known about the mechanisms of fungal response to the presence of quinoline and its biodegradation by fungi. Microorganisms from the genus *Curvularia* are microscopic fungi most often isolated from the soil, although they can also be isolated from plant and animal tissues [9,10]. Strains from this genus are able to undergo steroid transformation, produce compounds with antibacterial and antifungal activity, and degrade polyaromatic hydrocarbons, dyes, and polyethylene [11,12,13,14,15].

In the present work, the influence of quinoline on fatty acid and phospholipid profiles of *Curvularia lunata* IM 4417 was assessed. Additionally, the ability of *C. lunata* IM 4417 to degrade and detoxify quinoline was determined using toxicity tests based on organisms from different trophic levels. It is worth mentioning that the *C. lunata* IM 4417 strain has been previously described as a fungus that is able to 11β-hydroxylate cortexolone and degrade anthracene, phenanthrene, and organotin compounds [16,17,18].

## 2. Results

### 2.1. Biodegradation and Detoxification of Quinoline by C. lunata IM 4417

**Figure 2:**
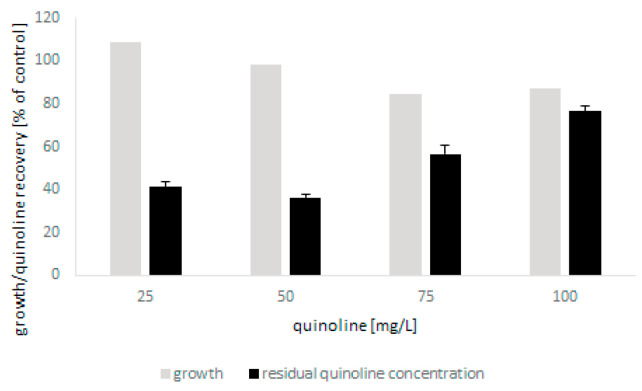
The growth of *C. lunata* IM 4417 in the presence of quinoline and its elimination during 48 h of incubation.

The tests showed that the microscopic fungus was able to grow in the presence of quinoline up to 100 mg/L without a significant limitation of biomass production. The biodegradation of N-heterocyclic compound was found to be closely correlated with the initial concentration of quinoline and was 60% and 20% for the lowest and highest concentrations, respectively (Figure 2). The conducted analyses also showed that bioconversion of quinoline led to the formation of small amounts of 2-hydroxyquinoline. Moreover, detailed chromatographic analysis revealed that during biodegradation, conjugates of glucose and quinoline and its hydroxylated derivatives were formed (Table 1). In the present work, three different glucose and hydroxyquinoline conjugates were determined, which may indicate that metabolites hydroxylated in various positions were formed during bioconversion of the substrate.

**Figure 3:**
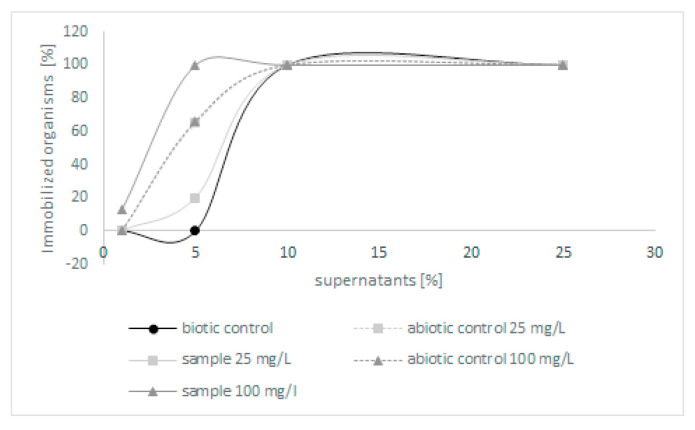
The toxicity of abiotic controls and post-culture liquids obtained after 48 h of incubation of *C. lunata* IM 4417 with quinoline.

**Figure 4:**
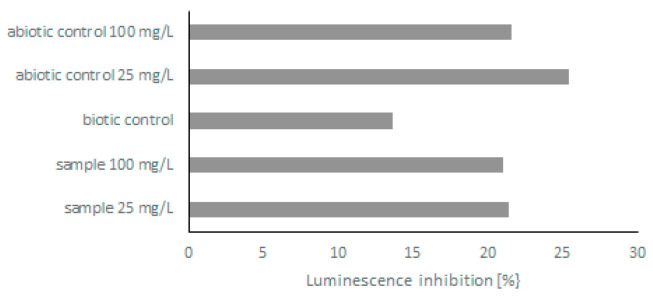
Bioluminescence reduction by abiotic control and post-culture liquids obtained after 48 h of incubation of *C. lunata* IM 4417 with quinoline.

Because few derivatives were formed during the biodegradation of quinoline, in the next step, the toxicity of post-culture liquids was assessed. The ability of *C. lunata* IM 4417 to detoxify quinoline was determined using two different Toxkits: Artoxkit M and Microtox assay. Based on the results obtained from the Artoxkit assay, it can be stated that the fluids obtained after the incubation of *C. lunata* IM 4417 with 25 mg/L quinoline showed much lower toxicity than the corresponding abiotic controls. Such a relationship was not found for the probes containing the highest tested concentrations of N-heterocyclic compounds (Figure 3). Additionally, the Microtox test showed that, in the samples containing 25 mg/L quinoline and *C. lunata* IM 4417, the inhibition of luminescence was lower than that in analogous abiotic controls. In the case of samples containing 100 mg/L of N-heterocyclic compound, the toxicity levels of samples with fungus and abiotic control were the same (Figure 4).

### 2.2. Modification of Fatty Acid Profile and Phospholipids of C. lunata IM 4417 during Incubation with Quinoline

**Figure 5:**
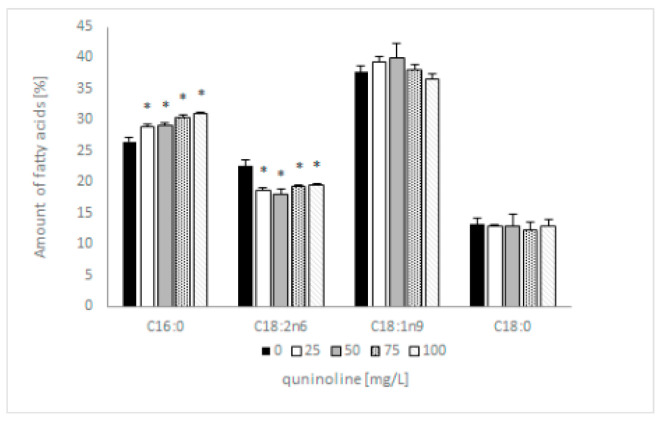
Influence of quinoline on the fatty acid profile of *C. lunata* IM 4417 determined in the stationary phase of growth. Asterisk (*p* < 0.05) indicates values that differ significantly from the control.

Previous research showed that 4 out of 12 identified fatty acids constitute 95% of total lipids and can be considered dominant. These were two saturated (C16:0; C18:0) and two unsaturated fatty acids (C18:1n9; C18:2n6). The addition of quinoline does not affect the type of synthesized fatty acids but influences the content of individual acids. In samples containing 100 mg/L quinoline, the content of C16:0 increased by 5% and C18:2 decreased by 3% in comparison with the control (Figure 5). The amount of C18:1 in the tested samples depended on the quinoline concentration, whereas the content of C18:0 remained constant regardless of the concentration of quinoline. The discussed changes influenced the value of the saturation index, which increased from 0.66 to 0.78 for the biotic control and culture with 100 mg/L quinoline. To expand our knowledge regarding the effects of quinoline in *C. lunata* IM 4417, phospholipid analysis of the mycelium was performed in the next set of studies.

In the present work, the following phospholipids (PLs) were analyzed: phosphatidic acid (PA), phosphatidylcholine (PC), phosphatidylethanolamine (PE), phosphatidylinositol (PI), phosphatidylserine (PS), and phosphatidylglycerol (PG). Among the analyzed PLs, PC and PE played dominant roles, and their sum was approximately 80% in the control samples (Table 2). On the basis of the obtained results, it can be concluded that the addition of quinoline essentially affects the two most important groups of phospholipids, i.e., PE and PC. In samples containing 25 mg/L quinoline, a four-fold decrease in the content of PA and a two-fold increase in the level of PC were observed. At the same time, a more than six-fold reduction in PI and PS content was noted. Additionally, it was found that the content of PE also decreased in the mentioned samples. Similar changes were observed in all probes containing quinoline, which resulted in a modification of the PC/PE ratio. The mentioned index reached values of 0.52, 1.51, and 2.48 for the control, the sample containing 25 mg/L quinoline, and the probe with 100 mg/L quinoline, respectively.

**Figure 6:**
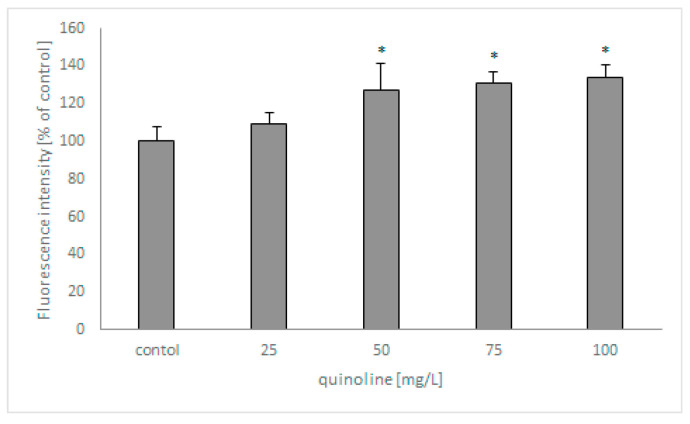
The effect of quinoline on the membrane permeabilization of *C. lunata* IM 4417. Asterisk (*p* < 0.05) indicates values that differ significantly from the control.

In the next set of studies, the influence of quinoline on fungal membrane permeabilization was determined using SYTOX Green (Figure 6). The analysis revealed that the fluorescence of the samples increased with increasing concentrations of quinoline and reached 135% in samples containing the studied compound at 100 mg/L.

## 3. Discussion

Quinoline, a typical N-heterocyclic compound, is one of the main pollutants formed during the processing of creosote and coal tar. Its good solubility, high toxicity, and low biodegradability has made it a serious threat to humans and nature. Despite the widespread presence of quinoline in the environment, the knowledge of its effects on fungi and their metabolism is still limited. Due to the fact that fungi are considered as useful models of mammalian metabolism, the *Curvularia lunata* strain was selected for the study. Abundant data in the literature indicate that fungi from the genus *Curvularia* have the ability to degrade various xenobiotics, such as phenanthrene, fluorene, or organotin compounds [17,18]. The present study focuses not only on the ability of *Curvularia lunata* IM 4417 to degrade and detoxify quinoline but also on adaptive changes occurring in the presence of the N-heterocyclic compound. Importantly, in the present work, quinoline concentrations typical for wastewater and ground water contaminated by creosote were used.

Many types of bacteria are characterized by high tolerance to quinoline and have the ability to degrade it, including *Pseudomonas*, *Rhodococcus*, *Streptomyces*, *Comamonas*, *Burkholderia*, and *Bacillus* strains [19,20,21,22]. The bacterial degradation of quinoline carried out under aerobic conditions is based on numerous hydroxylation reactions leading to the formation of dihydroxy derivatives and the breakdown of the rings that build the quinoline molecule. Moreover, literature data confirm that the first metabolite that is formed, regardless of the type of pathway, is 2-hydroxyquinoline.

Among microscopic filamentous fungi, only a few have been reported to grow in the presence of quinoline and have been described as capable of its elimination. Moreover, information on the biodegradation of quinoline by fungi is scarce. Similarly, little is known about the metabolites that are formed during this process. Literature data indicate that the ability to grow in the presence of quinoline is demonstrated by the white rot fungus *Pleurotus ostreatus* and *Cunninghamella elegans* 21Gp strain [23,24]. Additionally, analysis of extracts from *P. ostreatus* culture showed the presence of two unidentified metabolites of quinoline. In the case of *C. elegans* 21Gp, studies revealed that the tested microorganism converted quinoline into two hydroxylated derivatives: 2-hydroxyquinoline and 3-hydroxyquinoline. Sutherland et al. [25] also showed that a strain of *C. elegans* was capable of performing N-oxidation of quinoline.

The conducted analyses revealed that *C. lunata* IM 4417 shows a high tolerance in relation to quinoline and has the ability to eliminate the studied compound. The highest removal of quinoline was noted in samples containing up to 50 mg/L of quinoline, and in these probes, no inhibition of biomass production was observed. In addition, during the biodegradation process, trace amounts of hydroxylated derivatives of quinoline and three conjugates of hydroxyquinoline with glucose were formed. The mentioned metabolites confirm that during the degradation of quinoline by *C. lunata* IM 4417, hydroxylation of the studied compound first occurs, which may not be confined to the C-2 position. In the analyzed samples, N-glucose-quinoline was also identified. The presented data are in accordance with the conclusions presented in the paper of Felczak et al. [24] and confirm the fact that the hydroxylation of the tested compound may take place at several positions in the ring. Moreover, to our knowledge, this is the first paper describing the ability of microscopic filamentous fungi to produce hydroxyquinoline-glucose conjugates.

N-heterocyclic compounds, including quinoline, due to the presence of a nitrogen atom in their structure, are characterized by greater water solubility, mobility, and bioavailability than their homocyclic analogues. These features favor the accumulation of the mentioned substance in groundwater and soil, which in turn leads to increased exposure to quinoline organisms representing various trophic levels [6,26]. Because different derivatives are formed during the degradation of quinoline, including a few conjugates with glucose, in the next stage of studies, the toxicity of post-culture liquids was assessed. The research was carried out on the marine crustacean *Artemia franciscana* and bacteria *Aliivibrio fischeri* using the Artoxkit M and Microtox assays, respectively. Both tests are commonly used to determine the toxicity of various chemicals, waters, and wastewater. Additionally, the use of the mentioned toxicity models enabled us to obtain information about the influence of quinoline on different trophic levels.

Analysis of post-culture fluids containing 25 mg/L of quinoline using Artoxkit M showed that they were less toxic than the corresponding abiotic control. In these samples, good mycelium growth and high quinoline elimination was accomplished with the production of glucose conjugates with quinoline derivatives. This indicated that degradation of quinoline leads to detoxification of the N-heterocyclic compound. Additionally, the Microtox assay indicated that samples containing the lowest concentration of N-heterocyclic compound after incubation with *C. lunata* IM 4417 exhibited lower toxicity. To our knowledge, this is the first report showing the ability of *C. lunata* IM 4417 to degrade and detoxify quinolone.

It is well known that quinoline and its derivatives are ecotoxic compounds. Neuwoehnet et al. [6] found that the experimental EC50 values in *Daphnia* and *Aliivibrio fischeri* tests for quinoline reached 31.8 and 6.7 mg/L, respectively. The same authors also showed that the EC50 for 2-hydroxyquinoline was 63.4 mg/L for the *Daphnia* test and 0.9 mg/L for the *Aliivibrio fischeri* assay. Oberoi and Philip [27] studied the toxicity of quinoline and its hydroxylated derivatives and indicated that these compounds caused acute toxicity in *E. coli* and *P. fluorescence* tests. The EC50 values in the *P. fluorescence* assay were 49.4 and 75.0 mg/L for quinoline and 2–hydroxyquinoline, respectively. The literature data also indicate that biotransformation of quinoline does not always have to be associated with toxicity reduction, and hydroxylated derivatives may show even higher toxicity than parent compounds [6].

Because many toxic substances present in the environment interfere with components of cell structures, the fatty acid composition and the phospholipid profile are extremely important ecological indicators. Among membrane lipids, glycerophospholipids are the largest group and constitute approximately 70% of total membrane lipids. They are extremely sensitive to any changes in the environment and are responsible for the development of adaptations to various stress factors. PLs are the main structural components of the cell membrane, and they determine its stability, fluidity, and proper functioning. Some of them may also be involved in signal transduction or secretion [28].

The properties of the membrane are determined by the type of fatty acids, PL profile, acyl chain composition, and saturation index, and they are responsible for its proper functioning. The presented results indicate that the addition of quinoline to *C. lunata* IM 4417 causes a reduction in the C:18:2 content, with a simultaneous increase in the amount of C:16:0. Because linoleic acid is considered one of most important biomarker fatty acids in fungi [29], the significant decrease in C:18:2 content seems to be very important in an ecological context. Changes in fatty acids influenced the saturation index, which increased by 20%.

Literature data indicate that the presence of other xenobiotics can cause similar changes. Bernat and Długoński [30] indicated that the addition of tributyltin to *C. elegans* 21 Gp culture increases the content of saturated fatty acids. Additionally, studies investigating the influence of octyltin on the fatty acid profile of *C. lunata* showed that the presence of organotin compounds causes a decrease in the unsaturation index [18]. On the other hand, salt stress can cause both an increase in the content of unsaturated fatty acids or a decrease, depending on the strain tested, as demonstrated by Turk et al. [31], who studied yeast-like fungi.

Analysis of PL composition indicated that the main lipids in *C. lunata* IM 4417 biomass are PCs and PEs. This result is in accordance with published data, which show that the mentioned lipids account for over 50% of all PLs in fungi [32,33]. Both PC and PE are major structural lipids and determine membrane fluidity, wherein PCs are typical bilayer lipids and PEs are nonbilayer molecules. On the basis of the presented results, it can be concluded that the addition of quinoline causes an increase in PC content and a decrease in PE amount. These PLs are crucial for vegetative fungal growth [33]. Thus, this change may be essential to maintaining the proper functioning of the cell in the presence of quinoline and can lead to increased membrane fluidity. This hypothesis is confirmed by the results obtained in the SYTOX Green test, which has also shown that in the presence of quinoline, the membrane permeability increases. Increased membrane permeabilization in fungal cells was also shown in the presence of the antimicrobial peptide Pv D1. Moreover, a change in membrane permeability was closely correlated with fungal growth inhibition [34].

In samples containing quinoline, decreases in the contents of PIs and PSs were also noted. PIs are considered signal molecules and are a precursor in the synthesis of sphingolipids. Published data indicated that the decrease in the content of these lipids in *Saccharomyces cerevisiae* reduced cell viability. Additionally, PS are believed to play a crucial role in growth and tolerance to toxic compounds in some yeast strains [35].

In conclusion, understanding the influence of various xenobiotics on the PL profile seems to be crucial for understanding the adaptive mechanisms of fungi and may be an important indicator of the state of the environment in which these microorganisms live.

## 4. Materials and Methods

### 4.1. Microorganism

The filamentous fungus *Curvularia lunata* IM 4417, from the Culture Collection of the Department of Industrial Microbiology and Biotechnology, University of Lodz, Lodz, Poland, was used in the study.

### 4.2. Preparation of Inoculum

The spores from 14-day-old cultures on ZT slants were washed with 5 mL of Sabouraud medium (Difco) and transferred to 10 mL of fresh Sabouraud medium. The obtained inoculum was incubated at 28 °C on a rotary shaker (140 rpm) for 48 h.

### 4.3. Dry Weight Assessment

Biomass production was determined by filtering the samples through Whatman filter no. 1 and drying the mycelium at 105 °C to obtain a constant weight.

### 4.4. Lipid Profile Determination

Mycelium obtained after filtration was mixed with 10 mL of methanol and homogenized on a Fast-Prep-24 h Instrument (MP Biomedicals, Eschwege, Germany). In the next step, the supernatant was collected, and after supplementation with 2 mL of 0.9% NaCl, it was extracted with chloroform. The organic phase was collected, evaporated, dissolved in a methanol/chloroform mixture (4:1, *v*/*v*), and analyzed on an Agilent 1200 HPLC system with a 3200 QTRAP (Sciex Framingham, MA, USA) mass spectrometer [28].

### 4.5. Elimination of Quinoline

The preparation of samples included transfer of 2 mL of inoculum to 18 mL of fresh Sabouraud medium containing appropriate amounts of quinoline (concentrations ranged from 25 to 100 mg/L). Additionally, biotic controls (without xenobiotics) and abiotic controls (without inoculum) were prepared. All samples were incubated on a rotary shaker (140 rpm) at 28 °C for 48 h.

### 4.6. Quinoline Determination

Quinoline and its derivative contents were analyzed using gas chromatography–mass spectrometry (GC/MS) and liquid chromatography–tandem mass spectrometry (LC-MS/MS). A detailed description of the sample preparation and chromatographic methods is included in Felczak et al. [24]. Additionally, qualitative analysis involving the loss of the neutral glucose molecule (162 Da) was carried out using an Eksigent microLC 200 System (Sciex Framingham, MA, USA) connected with an AB Sciex 4500 QTRAP mass spectrometer (Sciex Framingham, MA, USA). A C18 column (50 mm × 0.5 mm, particle size: 2.7 μm, (Sciex Framingham, MA, USA)) maintained at 45 °C was used for the chromatographic separation of quinoline metabolites. The eluents with a constant flow of 15 μL/min were water (A) and acetonitrile (B). Both eluents were supplemented with 0.1% formic acid. The injection volume was 5 µL. The mass spectra of quinoline metabolites were collected at collision energy settings of 30 ± 15 and 50 ± 10 V. The gradient of eluents and the microESI ion source parameters are presented in Table 3. The negative mode was retained for quinoline metabolite analysis. Analyst™ version 1.6.2 software (Sciex Framingham, MA, USA) was used in the qualitative analysis of quinolone metabolites.

### 4.7. Artoxkit M

The Artoxkit M assay, which is based on *Artemia franciscana* crustaceans, was used to determine the ability of *C. lunata* IM 4417 to detoxify quinoline. The fluids from 48 h cultures of fungus containing 25 mg/L and 100 mg/L quinoline were filtered and analyzed. In addition, the toxicities of the parent abiotic and biotic controls were determined. The test was performed according to the standard ASTM E1440-91.

### 4.8. Microtox Assay

Assessment of *C. lunata* IM 4417 post-culture liquids toxicity was performed using the *Aliivibrio fischeri* DSM 7151 test in accordance with ISO 11348-1: 2007. The mentioned test allowed us to determine the effect of a selected xenobiotic on bioluminescence emissions, which in turn is closely related to cellular respiration and metabolism. The study was carried out with the use of post-culture fluids, obtained after biomass separation from samples containing 25 mg/L and 100 mg/L of quinoline, appropriate abiotic controls, and biotic probes. Post-culture fluids were diluted so that their final concentration was 1%.

### 4.9. Estimation of Fungal Membrane Permeability

The influence of quinoline on fungal membrane permeability was determined using a SYTOX Green assay. SYTOX Green nucleic acid stain is a substance that penetrates through damaged cell membranes. The spores of *C. lunata* IM 4417 were washed from Sabouraud slants, resuspended to a final concentration (5 × 10^5^ spores/mL) in Sabouraud medium, and supplemented with quinoline. After incubation (48 h at 28 °C), SYTOX Green was added to the studied samples, and the probes were incubated for 15 min in the dark. The fluorescence of fungal biomass was measured on a FLUOstar OMEGA at an excitation of 485 nm and emission of 535 nm. The obtained results are expressed as the percentage of fluorescence of the biotic control.

### 4.10. Statistical Analysis

All experiments were performed at least in triplicate and were analyzed individually. One-way analysis of variation was used to estimate the statistical significance of the results.

## 5. Conclusions

In summary, the conducted analyses showed that *C. lunata* IM 4417 has the ability to eliminate quinoline, and this process is most efficient at low concentrations. Quinoline bioconversion leads to the formation of conjugates of hydroxylated derivatives of the tested compound and glucose, as well as to a reduction in the toxicity of post-culture liquids. Furthermore, the addition of quinoline fundamentally influenced the fatty acid content of the tested strain. The presence of the compound affects the content of linoleic acid, which is an important ecological indicator and significantly modifies the phospholipid profile of *C. lunata* IM 4417. The addition of quinoline affects the content of PC and PE, two groups of lipids responsible for the proper functioning of the membrane and vegetative growth. These changes led to an increase in membrane permeability, which was confirmed by the SYTOX Green assay. Moreover, the changes in the content of PI and PS, which play a crucial role in signal transduction, cell viability, and tolerance to toxic compounds, were noted in the presence of quinoline.

## Figures and Tables

**Table 1:** LC-MS/MS results of quinoline biotransformation by *C. lunata* IM 4417.

Compound	Retention Time [min]	Chemical Formula	Metabolite Name	Metabolite Mass (Da)	Mass Spectrum *m*/*z* (Relative Intensity of 8 Largest Ions)
M1	4.73	C_15_H_17_NO_6_	Glucose-hydroxyquinoline	307	102.96 (28.1), 128.88 (41.7), 130.90 (100), 144.96 (12.5), 172.90 (23.9), 174.84 (63.5), 218.88 (17.7), 306.24 (8.3)
M2	5.48	C_15_H_17_NO_6_	Glucose-hydroxyquinoline	307	102.96 (31.3), 128.91 (31.3), 130.92 (100), 150.96 (18.8), 174.84 (31.3), 174.96 (31.3), 209.16 (25), 306.24 (84.4)
M3	6.00	C_15_H_17_NO_6_	Glucose-hydroxyquinoline	307	102.96 (25), 129.00 (43.8), 129.36 (12.5), 130.92 (100), 147.00 (31.3), 174.96 (100), 218.76 (12.5), 306.24 (43.8)
M5	6.82	C_15_H_17_NO_5_	N-glucose-quinoline	291	128.88 (31.6), 130.92 (100), 174.96 (73.7), 218.90 (36.8), 244.7 (42.1), 245.04 (21), 246.84 (42.1), 290.76 (47.4)

**Table 2:** The percentage of total phospholipids and their characteristic index values. Asterisk (*p* < 0.05) indicates values that differ significantly from the control.

Lipid Species	Control	Quinoline Concentration [mg/L]			
		25 mg/L	50 mg/L	75 mg/L	100 mg/L
PA	1.07 ± 0.24	0.25 ± 0.05 *	0.31 ± 0.02 *	0.34 ± 0.03 *	0.87 ± 0.17
PC	27.65 ± 4.48	58.32 ± 3.94 *	64.77 ± 4.22 *	72.21 ± 2.81 *	66.21 ± 5.45 *
PE	52.45 ± 9.93	38.57 ± 2.77 *	31.90 ± 4.51	23.58 ±1.62 *	26.65 ± 5.98 *
PI	16.40 ± 4.59	2.47 ± 0.81	2.55 ± 0.35	3.54 ± 1.03	5.56 ± 0.38
PS	2.43 ± 0.62	0.39 ± 0.31 *	0.47 ± 0.07 *	0.33 ± 0.06 *	0.72 ± 0.03
PC/PE	0.53 ± 0.09	1.51 ± 0.21 *	2.03 ± 4.42 *	3.06 ± 0.34 *	2.48 ± 0.59

**Table 3:** Eluent gradient and the parameters of the ion source used during LC-MS/MS analysis.

The Gradient of Eluents			The MicroESI Ion Source Parameters	
Time [min]	Eluent A [%]	Eluent B [%]		
0.0	98	2	**Parameter**	**Value**
0.2	98	2	Curtain gas	25 psi
7.0	2	98	Ion spray voltage	−4500 V
8.0	2	98	Temperature	400 °C
8.2	98	2	Gas 1	20 psi
8.6	98	2	Gas 2	30 psi

## Data Availability

The data presented in this study are available on request from the corresponding author.

## Sample Availability

Samples of the compounds are not available from the authors.
